# A Comparative Overview of the Role of Human Ribonucleases in Nonsense-Mediated mRNA Decay

**DOI:** 10.3390/genes15101308

**Published:** 2024-10-10

**Authors:** Paulo J. da Costa, Juliane Menezes, Raquel Guedes, Filipa P. Reis, Alexandre Teixeira, Margarida Saramago, Sandra C. Viegas, Cecília M. Arraiano, Luísa Romão

**Affiliations:** 1 Department of Human Genetics, National Institute of Health Dr. Ricardo Jorge, 1649-016 Lisbon, Portugal; pjgomes@unistra.fr (P.J.d.C.); julmenezes@ciencias.ulisboa.pt (J.M.); raqueldpguedes@gmail.com (R.G.); alexandre.teixeira.mgf@gmail.com (A.T.); 2 BioISI-Biosystems & Integrative Sciences Institute, Faculty of Sciences, University of Lisbon, 1749-016 Lisbon, Portugal; 3 Instituto de Tecnologia Química e Biológica António Xavier, Universidade Nova de Lisboa, 2780-157 Oeiras, Portugal; anafilipareis@gmail.com (F.P.R.); margaridasaramago@itqb.unl.pt (M.S.); sviegas@itqb.unl.pt (S.C.V.)

**Keywords:** mRNA surveillance, quality control, nonsense-mediated mRNA decay (NMD), nonstop decay (NSD), mRNA degradation, natural NMD targets

## Abstract

Eukaryotic cells possess surveillance mechanisms that detect and degrade defective transcripts. Aberrant transcripts include mRNAs with a premature termination codon (PTC), targeted by the nonsense-mediated decay (NMD) pathway, and mRNAs lacking a termination codon, targeted by the nonstop decay (NSD) pathway. The eukaryotic exosome, a ribonucleolytic complex, plays a crucial role in mRNA processing and turnover through its catalytic subunits PM/Scl100 (Rrp6 in yeast), DIS3 (Rrp44 in yeast), and DIS3L1. Additionally, eukaryotic cells have other ribonucleases, such as SMG6 and XRN1, that participate in RNA surveillance. However, the specific pathways through which ribonucleases recognize and degrade mRNAs remain elusive. In this study, we characterized the involvement of human ribonucleases, both nuclear and cytoplasmic, in the mRNA surveillance mechanisms of NMD and NSD. We performed knockdowns of SMG6, PM/Scl100, XRN1, DIS3, and DIS3L1, analyzing the resulting changes in mRNA levels of selected natural NMD targets by RT-qPCR. Additionally, we examined the levels of different human β-globin variants under the same conditions: wild-type, NMD-resistant, NMD-sensitive, and NSD-sensitive. Our results demonstrate that all the studied ribonucleases are involved in the decay of certain endogenous NMD targets. Furthermore, we observed that the ribonucleases SMG6 and DIS3 contribute to the degradation of all β-globin variants, with an exception for βNS in the former case. This is also the case for PM/Scl100, which affects all β-globin variants except the NMD-sensitive variants. In contrast, DIS3L1 and XRN1 show specificity for β-globin WT and NMD-resistant variants. These findings suggest that eukaryotic ribonucleases are target-specific rather than pathway-specific. In addition, our data suggest that ribonucleases play broader roles in mRNA surveillance and degradation mechanisms beyond just NMD and NSD.

## 1. Introduction

The accumulation of nonfunctional RNAs is detrimental to the cell and has been linked to several human diseases [1,2]. Therefore, RNA quality control processes play a crucial role in recognizing and eliminating aberrant RNAs [3,4,5,6,7]. One such surveillance mechanism is nonstop mRNA decay (NSD). NSD detects mRNAs that lack a translation termination codon. In these mRNAs, the ribosome is able to read-through into the 3′-poly(A) tail, but in the poly(A) stretch, the ribosome slows down and stalls. The stalling of the ribosome leads to ribosome collisions, which are detected by the NSD machinery. Subsequent ribosome ubiquitination at the interface of two collided ribosomes is considered the signal for mRNA decay, which occurs through 3′-5′ degradation by the exosome without prior deadenylation [4,8]. Conversely, transcripts with a premature translation termination codon (PTC) are detected and rapidly degraded by a different surveillance mechanism known as nonsense-mediated mRNA decay (NMD) [5,6]. By degrading mRNAs with nonsense codons, NMD prevents the synthesis of potentially deleterious C-terminally truncated proteins [5]. Interestingly, many physiological mRNAs, which possess features recognized by the NMD machinery, have also been identified as NMD substrates. This suggests an additional role for NMD as a posttranscriptional regulator of gene expression [9,10].

The degradation of NMD substrates involves both endonucleolytic and exonucleolytic activities [11]. Endonucleolytic degradation is catalyzed by SMG6, which cleaves the mRNA near the PTC. The resulting 5′ and 3′ RNA fragments are then quickly degraded by general cellular exonucleases [12,13]. In addition to the endonucleolytic route, mammalian cells possess alternative exonucleolytic decay pathways that involve deadenylation and decapping steps [5,11]. These pathways include SMG5-SMG7 or SMG5-PNRC2 proteins that further recruit the decapping complex (DCPC) and the deadenylation complex (CCR4-NOT) to remove the cap-binding complex and the poly(A) tail, allowing 5′-to-3′ and 3′-to-5′ RNA degradation by XRN1 and the RNA exosome, respectively [14,15,16,17]. Additionally, deadenylation-independent decapping and subsequent 5′-3′ degradation also contribute to NMD [5,14,15,16].

The exosome is a multiprotein complex responsible for major 3′-5′ RNA degradation in eukaryotes [18]. The eukaryotic core exosome is evolutionarily conserved and composed of 10 essential subunits found in both the nucleus and cytoplasm [19,20]. In yeast, only one of these subunits, DIS3, is responsible for the catalytic activity of the machinery [21,22]. As a member of the RNase II superfamily of exoribonucleases, DIS3 contains a conserved RNB domain responsible for the 3′-5′ exoribonucleolytic activity of the exosome [23,24]. Additionally, DIS3 has endonucleolytic activity conferred by its PIN domain [25,26]. In humans, two homologues are part of the exosome: DIS3 and DIS3-like exoribonuclease 1 (DIS3L1) [27,28]. Both DIS3 and DIS3L1 are active 3′-5′ exoribonucleases, but only DIS3 has endoribonuclease activity. Another difference between these two homologues is their subcellular localization. DIS3 primarily localizes in the nucleoplasm, with a small fraction in the cytoplasm, while DIS3L1 is exclusively localized in the cytoplasm [28].

DIS3L2, another member of the RNase II superfamily, is distinct from its counterparts. Unlike DIS3 and DIS3L1, DIS3L2 is not part of the exosome complex and is associated with an uridylation-dependent RNA degradation pathway [29,30,31,32,33,34]. Our lab has published a report on DIS3L2’s role in the decay of natural NMD targets in a transcript-specific manner [35]. The involvement of DIS3L2 in NMD has also been reported by others [36].

Our understanding about the involvement of different human DIS3 exosome-associated homologues in RNA surveillance mechanisms remains limited. To investigate this, we evaluated the roles of DIS3 and DIS3L1, along with ribonucleases SMG6, PM/Scl100, and XRN1, in the degradation of natural endogenous NMD targets. Our findings indicate that each of these ribonucleases contributes to the decay of at least one endogenous NMD target, highlighting their target-specific roles in NMD. Additionally, we explored their roles in the degradation of various human β-globin gene variants representing NSD and NMD-sensitive mRNAs. Our results show that the ribonucleases tested are not restricted to a single mRNA decay pathway but also participate in the degradation of normal and/or NSD mRNAs, demonstrating a broader involvement in RNA turnover beyond NMD.

## 2. Materials and Methods

### 2.1. Plasmid Constructs

Each of the human β-globin gene variants βWT, β15, β26, and β39 was obtained as previously described [37,38,39]. The NSD-sensitive variant βNS (nonstop) was obtained by site-directed mutagenesis at three sites: a TAA to AAA mutation at the natural stop codon and TAA to AAA and TGA to AGA mutations in the 3′UTR to eliminate any in-frame stop codons.

### 2.2. Cell Culture, Plasmid, and siRNA Transfections

HeLa cells were grown in Dulbecco’s modified Eagle’s medium (DMEM) supplemented with 10% fetal bovine serum and transiently transfected as previously described [35,40] using Lipofectamine 2000 reagent (Invitrogen, Carlsbad, CA, USA). All siRNA sequences are available in Supplementary Table S1. Twenty-four hours later, cells were harvested for RNA and protein expression analysis.

### 2.3. Isolation of Total RNA and Protein Lysates

Cells were lysed in NP40 buffer supplemented with Proteinase and RNase inhibitors to obtain protein extracts [35,40], and total RNA was extracted using the Nucleospin RNA extraction II Kit (Macherey-Nagel, Düren, Germany) following the manufacturer’s indications.

### 2.4. Western Blot Analysis

Protein lysates were resolved on a 10% SDS-PAGE gel and transferred to polyvinylidene difluoride (PVDF) membranes (Bio-Rad, Hercules, CA, USA) following standard protocols. Membranes were probed with the following primary antibodies: mouse anti-α-tubulin (Sigma-Aldrich, Saint Louis, MO, USA; loading control) at 1:50,000 dilution, rabbit anti-XRN1 (Novus Biologicals, Centennial, CO, USA) at 1:500 dilution, rabbit anti-DIS3 (Sigma-Aldrich, Saint Louis, MO, USA) at 1:250 dilution, and rabbit anti-DIS3L1 (Sigma-Aldrich, Saint Louis, MO, USA) at 1:250 dilution. Subsequently, detection was carried out by incubating the membrane with the appropriate secondary antibodies: goat anti-rabbit horseradish peroxidase conjugate (Sigma-Aldrich, Saint Louis, MO, USA) diluted at 1:3000 and goat anti-mouse horseradish peroxidase conjugate (Bio-Rad) diluted at 1:4000 for α-tubulin, followed by enhanced chemiluminescence.

### 2.5. Reverse Transcription, Semi-Quantitative PCR (sqPCR), and Real-Time PCR (RT-qPCR)

First-strand cDNA was synthesized from total RNA using reverse transcriptase (NZYtech) according to the manufacturer’s instructions.

Semi-quantitative PCR for SMG6, PM/Scl100, and glyceraldehyde 3-phosphate dehydrogenase (GAPDH) cDNAs were performed under similar conditions: 3 μL of the RT product was amplified in a 50 μL reaction volume using 0.2 mM dNTPs, 15 pmol of each primer (Supplementary Table S2), 0.75 U of DreamTaq (Thermo Scientific, Waltham, MA, USA), and 1x PCR buffer (Thermo Scientific, MA, USA). The thermocycler conditions were 95 °C for 5 min, followed by 25–35 cycles of 94 °C for 30 s, 52–60 °C for 60 s, and 72 °C for 90 s, with a final extension of 72 °C for 5 min. Ten-microliter aliquots from each RT-PCR sample were analyzed by electrophoresis on 2% agarose gels.

RT-qPCR was performed with the ABI 7000 Sequence Detection System (Applied Biosystems, Foster City, CA, USA) using SYBR Green PCR Master Mix (Applied Biosystems), as previously described [35,40]. Ct values of the β-globin mRNA amplicons were compared to the respective βWT counterpart or to βWT at Luc siRNA conditions, as indicated in figures, and normalized with the reference amplicon Ct value. The forward and reverse primer sequences are available in Supplementary Table S2. Quantification was performed by the relative standard curve method and mRNA levels were determined by RT-qPCR using primers specific for each natural NMD target and for GAPDH. All values are normalized to GAPDH mRNA levels [standard deviations are shown (*n* = 3, at least)].

### 2.6. Statistical Analysis

Statistical significance was assessed using one-way and two-way ANOVA, as appropriate. Significance levels were defined as follows: * *p* < 0.05, ** *p* < 0.01, *** *p* < 0.001, and **** *p* < 0.0001. Results are presented as the mean ± standard deviation from a minimum of three independent experiments.

## 3. Results

### 3.1. Human Ribonucleases Modulate the mRNA Levels of Different Natural NMD Targets

**Figure 1:**
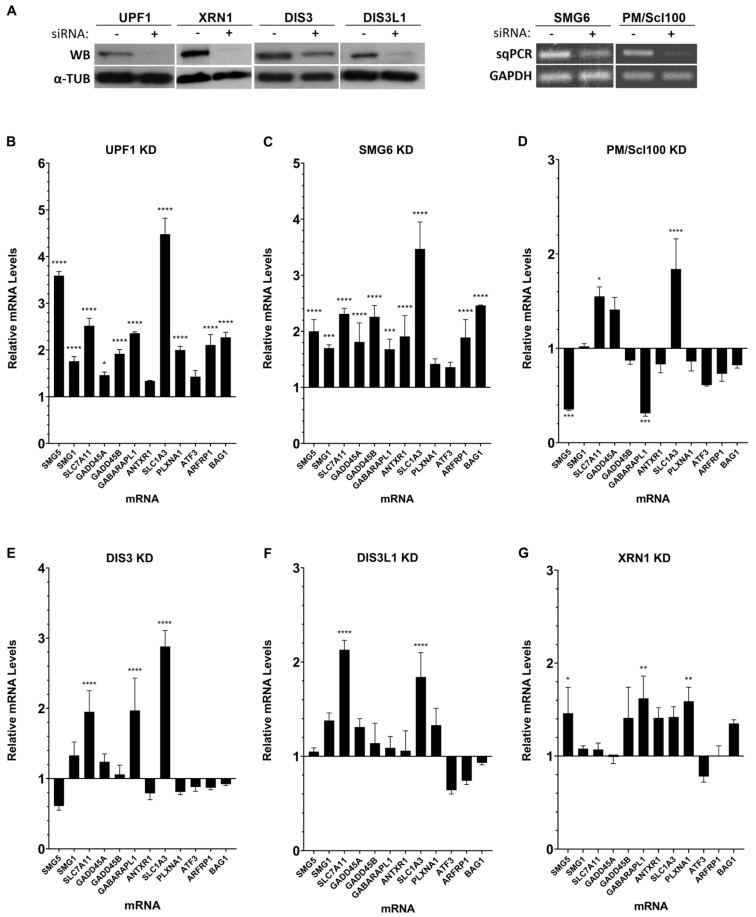
Human ribonucleases modulate the mRNA levels of different natural NMD targets. (**A**) Representative Western blot analysis of the UPF1, XRN1, DIS3, and DIS3L1 proteins and semiquantitative PCR analysis of the *SMG6* and *PM/Scl100* mRNA levels extracted from HeLa cells with (+) or without (−) knockdown (KD), to monitor KD efficiency. (**B**–**G**) Real-time PCR (RT-qPCR) analysis of natural NMD targets in HeLa cells transiently transfected with Luciferase (LUC) siRNA (Control), UPF1, SMG6, PM/Scl100, DIS3, DIS3L1, or XRN1 siRNAs. The *y*-axis represents fold change, with a value of 1 indicating no change relative to the control. Values above 1 indicate upregulation, while values below 1 indicate downregulation. The control (Luciferase KD) was normalized to a baseline of 1, and all other conditions are shown relative to this baseline. Asterisks (*) indicate statistical significance relative to the mRNA levels of the corresponding NMD target at control conditions: * *p* < 0.05, ** *p* < 0.01, *** *p* < 0.001, and **** *p* < 0.0001.

To study the impact of SMG6, PM/Scl100, DIS3, DIS3L1, or XRN1 on mRNA levels of natural NMD targets, we performed knockdowns (KDs) of each ribonuclease. As a positive control for NMD targeting, we knocked down UPF1, a key RNA helicase essential for initiation of the NMD pathway [41,42,43,44,45,46,47,48]. A siRNA targeting the luciferase mRNA sequence was used as a negative control (KD LUC). The KDs efficiency was monitored by sqPCR for SMG6 and PM/Scl100 and by Western blot analysis for UPF1, XRN1, DIS3, and DIS3L1 (Figure 1A). Subsequently, we assessed by RT-qPCR the effect of altering ribonuclease levels on the expression of selected natural NMD targets.

We selected 12 well-characterized natural NMD targets (*SMG5*, *SMG1*, *SLC7A11*, *GADD45A* and *B*, *GABARAPL1*, *ANTXR1*, *SLC1A3*, *PLXNA1*, *ATF3*, *ARFRP1*, and *BAG1*) and analyzed their mRNA levels in HeLa cells with and without depletion of ribonuclease genes (Figure 1). Fold-changes in mRNA levels are presented relative to control conditions (LUC siRNA-treated cells), which were arbitrarily set to 1.

Following the knockdown of the positive control gene UPF1, we observed a significant increase in mRNA levels for nearly all NMD targets studied, as expected, when compared to control conditions (Figure 1B).

Upon recognition by UPF1, NMD substrates can be degraded through various pathways. One known pathway involves the endonuclease SMG6, which, though primarily nuclear, also functions in the cytoplasm. SMG6 cleaves mRNA near the PTC, facilitating its degradation [12,13]. Depleting SMG6 resulted in substantial increases in the mRNA levels of nearly all analyzed natural NMD targets (Figure 1C). Specifically, *SMG5* and *SMG1* mRNA levels rose by approximately 2.0-fold and 1.7-fold, respectively. Both *SLC7A11* and *GADD45B* mRNA levels showed a 2.3-fold elevation, while *GADD45A* mRNA levels rose by 1.8-fold. *GABARAPL1* and *ANTXR1* mRNA levels elevated by 1.7-fold and 1.9-fold, respectively. Notably, *SLC1A3* mRNA increased substantially by 3.5-fold. *ARFRP1* accumulated by 1.9-fold and *BAG1* by 2.5-fold. This outcome was expected, as SMG6 is pivotal to the NMD process.

In the NMD pathway, after UPF1 initiates recognition and SMG6 induces endonucleolytic cleavage, the resulting mRNA fragments are targeted for subsequent exonucleolytic degradation from both the 5′ and 3′ ends. This degradation process involves recruiting decapping and 5′-to-3′ exonuclease activities, as well as deadenylating and 3′-to-5′ exonuclease activities.

The multiprotein complex exosome contains different catalytic subunits responsible for 3′-5′ exonucleolytic RNA degradation, including PM/Scl100 (Rrp6 in yeast), DIS3 (Rrp44 in yeast), and DIS3L1. Silencing the nuclear RNase PM/Scl100 influenced the mRNA levels of four out of the twelve NMD targets analyzed (Figure 1D). The mRNA levels of *SMG5* and *GABARAPL1* were reduced by 0.7-fold. In contrast, *SLC7A11* and *SLC1A3* exhibited an increment of about 1.6-fold and 1.8-fold, respectively. These results suggest that while PM/Scl100 does not regulate the majority of the NMD transcripts tested, it may affect specific mRNAs.

Upon depletion of DIS3, another catalytic subunit of the exosome primarily localized in the nucleus, *SLC7A11* and *GABARAPL1* mRNA levels increased by 2.0-fold, and *SLCA3* mRNA levels rose by 2.9-fold compared to the control (Figure 1E).

Furthermore, the mRNA levels of *SLC7A11* and *SLC1A3* increased by 2.1-fold and 1.8-fold, respectively, when the cytoplasmic exoribonuclease DIS3L1, also a member of the exosome complex, was knocked down (Figure 1F).

Distinct from the exosome-mediated 3′-5′ degradation, XRN1 is a key cytoplasmic enzyme involved in an alternative mRNA degradation pathway. As a 5′-3′ exonuclease, XRN1 plays a crucial role in general mRNA turnover by processing mRNAs from the 5′ end after decapping. Our study demonstrated that depletion of XRN1 led to the significant accumulation of *SMG5* mRNA by 1.5-fold and of both *GABARAPL1* and *PLXNA1* mRNAs by 1.6-fold (Figure 1G).

In this section, we investigated the effects of depleting various human ribonucleases on the mRNA levels of known endogenous NMD targets to elucidate their roles in the NMD mechanism. Notably, none of the ribonucleases tested exhibited a general effect on the NMD targets. Instead, they appear to be involved in the degradation of specific NMD targets, revealing target specificity. Additionally, the 3′-5′ exoribonucleases demonstrated redundancy over *SLC7A11* and *SLC1A13*, as both mRNAs can be degraded by all of them.

### 3.2. Roles of Human Ribonucleases in mRNA Surveillance Mechanisms: Use of Reporter Transcripts

**Figure 2:**
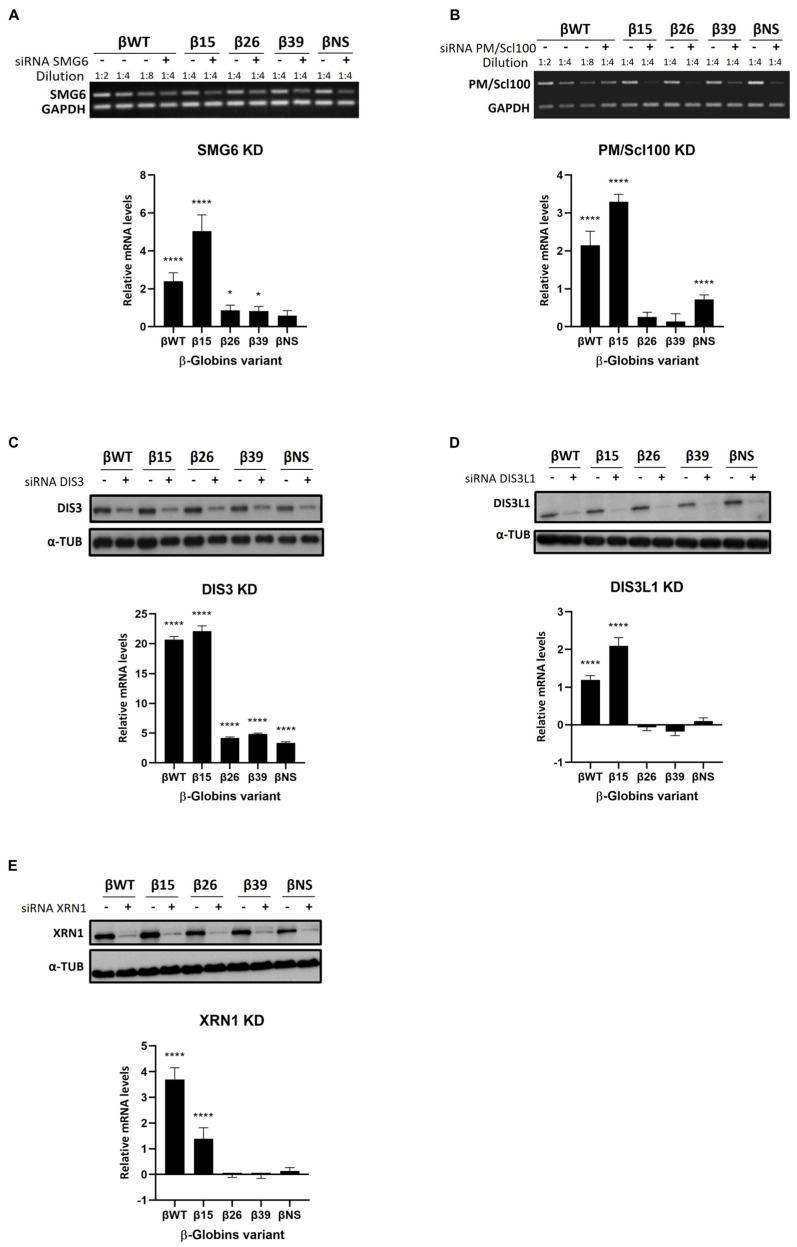
Human ribonucleases are involved in normal mRNA turnover as well as in the mRNA surveillance mechanisms of NMD and NSD. Representative semiquantitative PCR analysis of the *SMG6* (**A**) or *PM/Scl100* (**B**) mRNAs levels extracted from HeLa cells with (+) or without (−) knockdown (KD) and transiently transfected with plasmid-expressing wild type (βWT), NMD-resistant (β15), NMD-sensitive (β26 and β39), and NSD-sensitive (βNS) human β-globin mRNAs to monitor KD efficiency. (**C**–**E**) Representative Western blot analysis of protein extracted from HeLa cells with (+) or without (-) DIS3 and DIS3L1 KD and XRN1, respectively, and transiently co-transfected with plasmid-expressing βWT, β15, β26, β39, and βNS human β-globin mRNAs to monitor KD efficiency. (**A**–**E**) Real-time PCR (RT-qPCR) analysis of human β-globin mRNAs variants in HeLa cells transiently co-transfected with Luciferase (LUC) siRNA (Control), SMG6, PM/Scl100, DIS3, DIS3L1, or XRN1 siRNAs. The *y*-axis represents the fold change in mRNA levels. Values above 0 indicate upregulation, while values below 0 indicate downregulation. To compare the impact of each ribonuclease knockdown (KD) relative to the control, the fold change observed under Luciferase KD was subtracted from the fold change observed under each ribonuclease KD. This adjustment highlights the additional effect of the ribonuclease KD on mRNA variations compared to the control condition. Asterisks (*) indicate statistical significance relative to the mRNA levels of the corresponding NMD target under control conditions: * *p* < 0.05 and **** *p* < 0.0001.

To further elucidate the role of human ribonucleases in translation-dependent mRNA surveillance mechanisms such as NMD and NSD, we used the same genetic depletions as above: KD of SMG6, PM/Scl100, DIS3, DIS3L1, and XRN1 (Figure 2). We transiently co-transfected HeLa cells depleted on each of these ribonucleases with constructs containing different human β-globin variants. These variants contain nonsense mutations in different codons of the β-globin gene that make the transcript βWT (wild type) become NMD-resistant (β15), NMD-sensitive (β26 and β39), and NSD-sensitive (βNS) [37]. We assessed the changes in mRNA levels of all these different human β-globin variants by RT-qPCR.

We first analyzed the intrinsic effect in terms of mRNA accumulation levels of each of these variants under control conditions (LUC KD) (Appendix A). We observed a 1.3-fold increase in β15 mRNA levels relative to βWT (arbitrarily set to 1), suggesting evasion of NMD. In contrast, the NMD-sensitive transcripts, β26 and β39, exhibited significant reductions to 0.6-fold of βWT levels, indicating that these transcripts are targeted for degradation by the NMD surveillance mechanism. The βNS mRNA levels decreased 0.3-fold. Throughout all experiments, β-globin mRNA levels in control conditions (LUC KD) consistently exhibited the same pattern, demonstrating the reproducibility of our system.

When we tested the KD of SMG6 (Figure 2A) [12,13], we observed a significant increase in the mRNA levels of all β-globin variants, except βNS.

We also investigated the role of 3′-5′ exonucleases, which generally act after endonucleolytic cleavage events. Specifically, we examined the nuclear exonuclease PM/Scl100, a component of the exosome complex, by measuring the mRNA levels of human β-globin variants following its depletion (Figure 2B). Our results indicate that PM/Scl100 exhibits specificity for NMD-resistant and NSD-sensitive variants, with an increased mRNA fold-change variation for βWT (2.1-fold) and β15 (3.3-fold), as well as βNS (0.7-fold) (Figure 2B). In contrast, the NMD-sensitive variants β26 and β39 were unaffected.

We conducted similar experiments to investigate the role of DIS3, a predominantly nuclear 3′-5′ exonuclease, by measuring the mRNA levels of human β-globin variants following its KD (Figure 2C). Under these conditions, mRNAs of all β-globin variants, including βWT (20.7-fold), β15 (22.1-fold), β26 (4.2-fold), β39 (4.8-fold), and βNS (3.4-fold), increased significantly compared to control conditions (Figure 2C). These results suggest that DIS3 is not specific to any particular surveillance mechanism. However, its effect is notably more pronounced compared to other ribonucleases, leading to the largest increases in mRNA levels, particularly for the βWT and NMD-resistant variant (β15).

Knockdown of the cytoplasmic 3′-5′ exonuclease DIS3L1 resulted in elevated mRNA levels of β-globin variants βWT (1.2-fold) and β15 (2.1-fold) (Figure 2D). The levels of the NMD-sensitive variants β26 and β39 and of the NSD-sensitive variant, βNS, remained unchanged.

A similar pattern was observed following the knockdown of the 5′-3′ cytoplasmic exoribonuclease XRN1 (Figure 2E). After XRN1 KD, mRNA levels of the β-globin variants βWT (3.7-fold) and β15 (1.4-fold) increased significantly (Figure 2E). In contrast, XRN1 KD had minimal effects on the mRNA levels of the NMD-sensitive variants β26 and β39, as well as on the NSD-sensitive variant βNS, showing negligible differences from control levels (LUC KD). Our results suggest that both DIS3L1 and XRN1 exhibit some degree of specificity towards the βWT and the NMD-resistant variant β15.

In summary, our study using β-globin variants, encompassing both NMD and NSD reporters, demonstrates that ribonucleases SMG6 and DIS3 play significant roles in the degradation of all β-globin variants, with an exception for βNS in the former case. PM/Scl100 specifically affects β-globin WT, as well as NMD-resistant and NSD-sensitive variants. Conversely, DIS3L1 and XRN1 show particular specificity for β-globin WT and NMD-resistant variants.

## 4. Discussion

Eukaryotic cells have evolved various mRNA decay mechanisms to ensure proper mRNA processing and translatability. However, these mechanisms are more intricate than previously thought. Even within the well-studied mRNA quality control pathway of NMD, new details continue to emerge. The degradation of both normal and defective mRNAs is caused by ribonucleases, which are essential players in RNA decay processes. However, the specific roles and contributions of individual ribonucleases in these decay mechanisms remain unclear.

In this work, we investigated the roles of several human ribonucleases—SMG6, PM/Scl100, DIS3, DIS3L1, and XRN1—in the mRNA surveillance mechanisms of NMD and NSD. Building on our previous findings that depletion of DIS3L2 leads to significant accumulation of natural NMD targets [35,40], we aimed to further explore how these ribonucleases regulate the mRNA levels of 12 natural NMD targets: *SMG5*, *SMG1*, *SLC7A11*, *GADD45A*, *GADD45B*, *GABARAPL1*, *ANTXR1*, *SLC1A3*, *PLXNA1*, *ATF3*, *ARFRP1*, and *BAG1* [35,40,49] (data summarized in Table 1). Our study extended this investigation by also exploring their roles in aberrant mRNA turnover using a range of β-globin variants representing normal, NMD-sensitive, NMD-resistant, and NSD-sensitive mRNA forms (data summarized in Table 2). By comparing the effects of ribonuclease depletions on various targets, we aimed to uncover the mechanisms through which these proteins contribute to mRNA decay and surveillance processes.

All the genes tested in this work play pivotal roles in various cellular processes. SMG5 and SMG1 are core components of the NMD machinery, SMG5 being involved in the degradation of NMD targets and SMG1 acting as a kinase that phosphorylates UPF1, initiating the NMD process [50,51]. SLC7A11 and SLC1A3 are involved in amino acid transport and neurotransmitter cycling, respectively, and their expression is tightly regulated to maintain cellular homeostasis [52,53]. GADD45A and GADD45B are stress response genes that mediate DNA repair and cell cycle control, crucial for preventing genomic instability [54]. GABARAPL1 is implicated in autophagy [55], while ANTXR1 plays a role in cell adhesion and immune response [56]. PLXNA1 guides axon growth and cell migration, and ATF3 is a transcription factor involved in stress response [57]. ARFRP1 regulates intracellular trafficking [58], and BAG1 is a co-chaperone that influences cell survival and apoptosis [59]. NMD ensures the fidelity of gene expression in these natural mRNAs, since they have features that can induce premature translation termination. This degradation process controls protein levels according to cellular needs, preventing the production of abnormal amounts of protein that could disrupt cellular function and contribute to disease.

In general, surveillance mechanisms exist both in the nucleus and the cytoplasm to detect errors at all stages of mRNA production and maturation. In this study, we tested the involvement of the nuclear ribonucleases PM/Scl100 and DIS3, as well as the cytoplasmic ribonucleases DIS3L1 and XRN1, in these mechanisms. Additionally, we investigated SMG6, which functions in both the nucleus and cytoplasm.

The SMG6 has long been recognized as a crucial endoribonuclease in the NMD surveillance mechanism, often referred to as the “long sought NMD endonuclease” [12], a concept consistently supported by numerous studies [60,61,62,63]. SMG6 functions by cleaving mRNAs near PTCs, initiating the degradation process essential for NMD [61]. Our data show that SMG6 knockdown leads to increased mRNA levels for almost all the studied NMD targets (10 out of 12) (Figure 1; Table 1). These results not only reaffirm the well-documented function of SMG6 in the NMD pathway but also validate our experimental system. SMG6 KD also causes elevated mRNA levels for two of the β-globin variants, WT and NMD-resistant form β15 (Figure 2 and Table 2). This aligns with previous evidence showing that SMG6 is involved in the decay of mRNAs, even if they do not display NMD-inducing features [64,65]. Accordingly, Metze and colleagues (2013) demonstrated that SMG6 knockdown increases the mRNA levels of PTC-free reporter gene variants [64]. Additionally, Nicholson et al. (2014) found that tethering SMG6 to an mRNA lacking NMD features significantly reduces its mRNA levels [65].

In the nucleus, defective mRNAs arising from errors in transcription or maturation are targeted for degradation. Both 3′ to 5′ and 5′ to 3′ pathways contribute to nuclear mRNA turnover. The exosome complex, a critical player in this process, relies on ribonucleases DIS3 and PM/Scl100 for its 3′-5′ RNA degradation activity. During mRNA maturation in the nucleus, which includes splicing and the addition of a poly(A) tail, the exosome degrades improperly processed mRNAs, such as those that are un-spliced or have defective polyadenylation [6,7].

DIS3 is predominantly a nuclear ribonuclease (with some presence in the cytoplasm) and functions as both a 3′-5′ exoribonuclease and endoribonuclease within the nuclear exosome. Initially discovered in yeast for its role in ribosomal RNA (rRNA) processing [19], DIS3 and the exosome were later found to be involved in mRNA quality control mechanisms such as NMD and general mRNA turnover in yeast cells. However, its activity was reported to not be required for the degradation of NSD mRNAs [6,7,12,66,67,68,69]. It remains to be elucidated whether the DIS3 exosome component has a direct role in the degradation of mRNAs in mammalian cells, specifically those targeted by surveillance pathways. To address this, we investigated DIS3-depleted cells and found that only three of the measured mRNAs—*SLC7A11*, *GABARAPL1*, and *SLC1A3*—accumulated (Figure 1; Table 1). This suggests that most of the NMD targets tested do not require DIS3 for their degradation. Additionally, DIS3 knockdown substantially increased the mRNA levels of all β-globin variants, with a more pronounced effect on the WT and NMD-resistant β-globin variants (Figure 2; Table 2). The observation that the NMD- and NSD-sensitive variants are less affected aligns with the fact that both NMD and NSD are generally considered cytoplasmic processes, whereas DIS3 is predominantly localized in the nucleus. However, the depletion of DIS3 resulted in significantly higher expression levels of all β-globin variant mRNAs compared to the other ribonuclease knockdowns. In the nucleus, DIS3 is known to maintain RNA polymerase II transcriptome homeostasis and control widespread transcription initiation and premature termination [70]. Therefore, we attribute the observed effect to DIS3′s association with RNA polymerase II and its role in ensuring the rapid degradation of unwanted RNAs. This accumulation likely results from the buildup of these species, which can exceed the levels of mature mRNAs, thereby contributing to the observed mRNA increase [71,72]. Overall, our study indicates that while DIS3 does not play a major role in NMD, it is directly involved in the decay of certain specific NMD targets. Moreover, this ribonuclease appears to maintain normal levels of the β-globin mRNA reporters: WT and NMD-resistant.

PM/Scl100, also known as EXOSC10, is a nuclear ribonuclease with 3′-5′ exoribonucleolytic activity involved in the degradation of various RNA species, including normal and PTC-containing mRNAs, as well as structured RNAs such as rRNAs, small nuclear RNAs (snRNAs), and small nucleolar RNAs (snoRNAs) [14,73,74,75]. Our data indicate that PM/Scl100 has a limited impact on most natural NMD target mRNAs (Figure 1; Table 1). Exceptions include the increased mRNA levels of *SLC7A11* and *SLC1A3*, which may result from the inhibition of nuclear degradation of their corresponding pre-mRNAs, leading to transcript accumulation. Conversely, a reduction in mRNA levels was observed for certain NMD targets, with a more pronounced effect on *SMG5* and *GABARAPL1* mRNAs. This reduction likely reflects an indirect effect, as PM/Scl100 may influence other regulatory factors involved in controlling these mRNAs. Additionally, PM/Scl100 affects the mRNA levels of the βWT, the NMD-resistant β15 variant, and the NSD-sensitive βNS, while NMD-sensitive variants (β26 and β39) remain unaffected (Figure 2; Table 2). To our knowledge, this study provides the first evidence linking PM/Scl100 to the degradation of NSD targets. In yeast, the PM/Scl100 homolog Rrp6 acts as a channel gate, enhancing Dis3′s catalytic activity within the exosome’s central channel and stimulating both exo- and endoribonuclease activities of Dis3 [76,77,78]. Our findings suggest that the effects observed in WT β-globin and NMD-resistant variants are likely due to the combined inhibition of PM/Scl100 and the resultant decrease in DIS3 catalytic activity. However, this does not fully explain why NMD-sensitive mRNAs (β26 and β39) remain unaffected (Figure 2C). One possible explanation is that, despite reduced nuclear degradation of these NMD-sensitive variants due to PM/Scl100 silencing and diminished DIS3 activity, cytoplasmic surveillance mechanisms might compensate for the increased levels of these mRNAs that have been exported to the cytoplasm.

XRN1 is a major cytoplasmic 5′-3′ exoribonuclease responsible for degrading mRNAs following the removal of their 5′ cap, a process known as decapping, which makes the RNA susceptible to degradation. This ribonuclease is well-documented for its involvement in bulk mRNA degradation [79,80], as well as in the decay of intermediates in NSD and NMD [79,81,82]. Our study further reveals that XRN1 depletion affects the mRNA levels of 3 out of 12 tested natural NMD targets (*SMG5*, *GABARAPL1*, and *PLXNA1*) (Figure 1; Table 1). This finding confirms that XRN1 is involved in NMD target degradation. However, it does not appear to be essential for the decay of all NMD targets. The variability observed among different NMD targets could be attributed to specific features that either inhibit decapping or interfere with XRN1-mediated degradation, like what has been observed with some viral RNAs [83,84]. Additionally, XRN1 targets both βWT and NMD-resistant β-globin variants but does not significantly affect the NMD- and NSD-sensitive variants.

Unlike XRN1’s 5′-3′ degradation pathway, the exosome complex utilizes the alternative 3′-5′ degradation pathway for RNA decay, with different exosome-associated ribonucleases operating depending on the cellular location of the complex [40]. DIS3L1 is a cytoplasmic exosome component with 3′-5′ exoribonucleolytic activity. This ribonuclease has been primarily studied in yeast, with limited research in mammalian cells revealing its involvement in rRNA degradation and the accumulation of polyadenylated rRNA intermediates upon its depletion [27,28]. Our study advances current understanding by demonstrating that DIS3L1 plays a role in the decay of mRNAs encoding natural NMD targets, such as *SLC7A11* and *SLC1A3* (Figure 1; Table 1). We also observed that depleting DIS3L1 leads to increased mRNA levels for the WT β-globin and NMD-resistant (β15) variants (Figure 2; Table 2). Therefore, our findings reveal that DIS3L1 exhibits target specificity within the NMD pathway, affecting certain targets but not others. Also, this research is the first to provide evidence of DIS3L1’s involvement in specific RNA decay processes.

DIS3L2, unlike its DIS3 family counterparts (DIS3 and DIS3L1), is not part of the exosome and is involved in cytoplasmic RNA degradation pathways, particularly those involving uridylation-dependent RNA decay mechanisms. This process relies on the addition of untemplated uridine residues to mRNAs and other RNA classes by terminal uridylyl transferases (TUTases). Our previous research has shown that nearly half of the natural NMD targets tested in this study are substrates of DIS3L2 in human cells (Table 1) [35]. These targets include *SMG1*, *SLC7A11*, *GADD45A*, *GADD45B*, *SLC1A3*, *PLXNA1*, and *ATF3*. Consequently, among all the ribonucleases discussed in this work, DIS3L2 appears to have the most significant impact on NMD transcripts.

Overall, we found that human ribonucleases, whether localized in the nucleus and/or cytoplasm, exhibit selectivity for specific natural NMD targets. This selectivity appears to be influenced by the unique characteristics of each transcript, such as its sequence and length, G, C, A, and U relative content, secondary structure, etc. Despite target specificity, our findings also indicate the existence of functional redundancy among these ribonucleases. For instance, we observed that mRNA levels of *SLC7A11* and *SLC1A3* accumulate following the knockdown of the 3′-5′ exonucleases PM/Scl100, DIS3, DIS3L1, and DIS3L2. This functional redundancy might be due to a common feature observed in the different target transcripts. In contrast, these mRNAs are not affected by the 5′-3′ exonuclease XRN1. Additionally, we found that these ribonucleases are involved in the decay of normal mRNAs and, beyond NMD, participate in other surveillance pathways such as NSD. Together, our results show that ribonucleases act specifically on certain mRNA targets, but multiple ribonucleases function redundantly. The interplay between specificity and redundancy may be explained by the presence of single or multiple and exclusive or shared transcript features, respectively. As an example, the 3′-end uridylation of specific RNAs makes them Dis3L2 targets [29,30,31,32,33,34]. Nevertheless, a more detailed mechanistic understanding of this interplay is of great interest for investigation in the future.

## 5. Conclusions

Transcriptome-wide analyses have revealed that NMD modulates ∼10% of human cell mRNAs [9]. Understanding how ribonucleases regulate subsets of NMD targets could reveal new therapeutic approaches for diseases such as cancer, where NMD pathway dysregulation plays a significant role. The understanding of their involvement in NSD is also important, since it also plays a role in the etiology of some genetic disorders [85,86].

Our findings provide a comprehensive overview of how different human ribonucleases influence the stability of transcripts targeted by the surveillance mechanisms of NMD and NSD. In these pathways, they reveal some redundancy between them and act in a target-specific manner. We show that ribonucleases are not confined to specific surveillance pathways but play broader roles in mRNA surveillance and turnover.

## Figures and Tables

**Table 1:** Summary of the observed effects on mRNA levels of endogenous NMD targets obtained under each ribonuclease knockdown. ↑—increase, ↓—decrease, ↔—not affected.

		Ribonucleases				
Targets	SMG6 ^a^	PM/Scl100 ^a^	DIS3 ^a^	DIS3L1 ^a^	XRN1 ^a^	DIS3L2 ^b^
SMG5	↑	↓	↔	↔	↑	↔
SMG1	↑	↔	↔	↔	↔	↑
SLC7A11	↑	↑	↑	↑	↔	↑
GADD45A	↑	↔	↔	↔	↔	↑
GADD45B	↑	↔	↔	↔	↔	↑
GABARAPL1	↑	↓	↑	↔	↑	↔
ANTXR1	↑	↔	↔	↔	↔	↔
SLC1A3	↑	↑	↑	↑	↔	↑
PLXNA1	↔	↔	↔	↔	↑	↑
ATF3	↔	↔	↔	↔	↔	↑
ARFRP1	↑	↔	↔	↔	↔	↔
BAG1	↑	↔	↔	↔	↔	↔

^a^ this work; ^b^ [35].

**Table 2:** Summary of the observed effects on mRNA levels of the human β-globin variants obtained under each ribonuclease knockdown. (βWT) wild type, (β15) NMD-resistant, (β26 and β39) NMD-sensitive, and (βNS) NSD-sensitive. ↑—increase, ↔—not affected.

Targets	SMG6	PM/Scl100	DIS3	DIS3L1	XRN1
βWT	↑	↑	↑	↑	↑
β15	↑	↑	↑	↑	↑
β26	↑	↔	↑	↔	↔
β39	↑	↔	↑	↔	↔
βNS	↔	↑	↑	↔	↔

**Figure A1:**
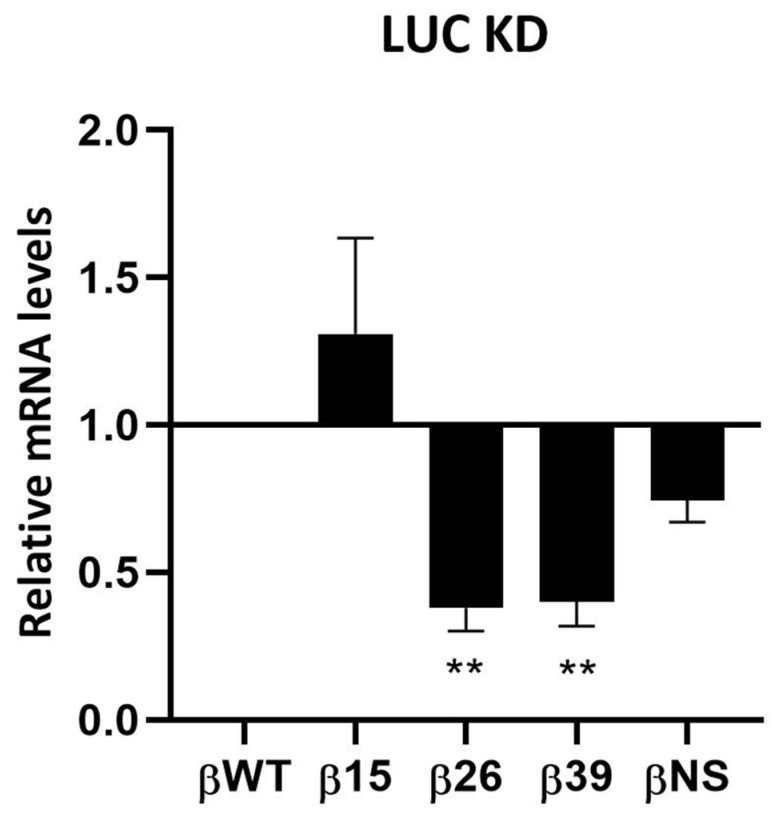
Real-time PCR (RT-qPCR) analysis of human β-globin mRNAs variants in HeLa cells transiently co-transfected with Luciferase (LUC) siRNA (Control). The y-axis represents mRNA fold change. The control (Luciferase KD) was normalized to a baseline of 1, and all other conditions are shown relative to this baseline. Values above 1 indicate upregulation, while values below 1 indicate downregulation. Asterisks (*) indicate statistical significance relatively to the mRNA levels of the corresponding NMD target at control conditions. ** *p* < 0.01.

## Data Availability

The data that support the findings of this study are available from the corresponding author upon reasonable request.

## Supplementary Materials

The following supporting information can be downloaded at https://www.mdpi.com/article/10.3390/genes15101308/s1. Table S1: siRNAs used in this work; Table S2: Oligonucleotides used in this work.
