# Supplementary material for: A Comparative Overview of the Role of Human Ribonucleases in Nonsense-Mediated mRNA Decay
Source: Genes (Basel). 2024 Oct 10;15(10):1308. doi: 10.3390/genes15101308 (PMC11507897; doi:10.3390/genes15101308)
Supplement: Supplementary file 1 [file genes-15-01308-s001.zip › genes-3207708-supplementary.pdf]

Supplementary Table S1- siRNAs used in this work

siRNA Target Gene	Sequence (5’→ 3’)
**Luciferase (LUC)**	CGUACGCGGAAUACUUCGA
**DIS3**	AGGUAGAGUUGUAGGAAUA
**DIS3L1**	CCAUGUAACCGUAAGAAUA
**XRN1**	GGGAUCUGGAAAGAUGCAAUACUUU
**PM/Scl-100 (RRP6)**	GCUGCAGCAGAACAGGCCA
**SMG6**	AAGCCAGUGAUACAGCGAA
**UPF1**	AAGAUGCAGUUCCGCUCCAUU

Supplementary Table S2- Oligonucleotides used in this work

Target	Orientation	Sequence
**HBB**	Forward	GTGGATCCTGAGAACTTCAGGC
**HBB**	Reverse	CAGCACACAGACCAGCACGT
**GAPDH**	Forward	CCATGAGAAGTATGACAACAGCC
**GAPDH**	Reverse	GGGTGCTAAGCAGTTGGTG
**SMG6**	Forward	GACACCAACGGCTTCATTGA
**SMG6**	Reverse	CAGGCCGTCCAGCTCATT
**PM/Scl100**	Forward	AGAGAGAGCGAGCAACAAGC
**PM/Scl100**	Reverse	TCCAGCAAAAGCCTTGAAGT
**SMG5**	Forward	CCCCTCATAGGATGCAAGAA
**SMG5**	Reverse	ATCTGTGCCCAATCCATCTC
**SLC7A11**	Forward	GGGCATGTCTCTGACCATCT
**SLC7A11**	Reverse	TCCCAATTCAGCATAAGACAAA
**GADD45A**	Forward	GGAGGAATTCTCGGCTGGAG
**GADD45A**	Reverse	CGTTATCGGGGTCGACGTT
**GABARAPL1**	Forward	GGCCAGTTCTACTTCTTAATCCGG
**GABARAPL1**	Reverse	AGGTGCTCCCATCTGCTGGG
**SMG1**	Forward	TGTGAGCAGGTTTTACACATTATGC
**SMG1**	Reverse	CCAGAGGGTCGTACACAAAGG
**GADD45B**	Forward	ACAGTGGGGGTGTACGAGTC
**GADD45B**	Reverse	GGATGAGCGTGAAGTGGATT
**SLC1A3**	Forward	TTCTCCTTTCCTGGGGAACT
**SLC1A3**	Reverse	CCATCTTCCCTGATGCCTTA
**DIS3L1**	Forward	CTTTTGTTCGACTTCAAGGAGCC
**DIS3L1**	Reverse	CCGTGGATTAGGATGTCACTGA
**ATF3**	Foward	ATCACAAAAGCCGAGGTAGC
**ATF3**	Reverse	TCTTGTTTCGGCACTTTGC
**ARFRP1**	Forward	CTAAACATCGGCACTGTGGA

**ARFRP1**	Reverse	ACTTGTCCCACAAAGACTGC
**BAG1**	Forward	TGGGAAAAAGAACAGTCCACA
**BAG1**	Reverse	TCCAGCTGGTCAGCTATCTTC
**ANTXR1**	Forward	ATGCCTTGTGGGTCCTACTG
**ANTXR1**	Reverse	GAGGTGTGGTAGGCGTTGTT
**PLXNA1**	Forward	GTGAAGAACCACGACCACCT
**PLXNA1**	Reverse	CGTGCTGAAGATGGTCTCAA
